# *Eucommia ulmoides* Oliv. and its bioactive compounds: therapeutic potential in bone diseases

**DOI:** 10.3389/fphar.2025.1601537

**Published:** 2025-06-24

**Authors:** Cai Huang, Huan Jin, Yan Zhang, Di Wang, Ziyi He, Bo Shuai

**Affiliations:** ^1^ Department of Integrated Traditional Chinese and Western Medicine, Union Hospital, Tongji Medical College, Huazhong University of Science and Technology, Wuhan, China; ^2^ College of Sports Medicine, Wuhan Sports University, Wuhan, China; ^3^ Department of Pain, Union Hospital, Tongji Medical College, Huazhong University of Science and Technology, Wuhan, China

**Keywords:** *Eucommia ulmoides* Oliv., bioactive compounds, aging-related bone diseases, bone formation, bone resorption, pharmacological mechanisms

## Abstract

**Background:**

Aging-related bone diseases encompass a range of conditions that emerge or worsen with advancing age, including osteoporosis and osteoarthritis, and they are placing an increasing burden on society. Although these diseases differ in clinical manifestations and pathological features, they often share common age-associated mechanisms such as impaired bone remodeling, chronic low-grade inflammation, cellular senescence, oxidative stress, and hormonal changes. Current therapies often face limitations in efficacy or long-term safety, highlighting the need for alternative strategies. Phytochemicals derived from Chinese medicine herb have emerged as promising candidates due to their multi-target effects on bone homeostasis. *Eucommia ulmoides* Oliv. (EU) and its bioactive compounds (e.g., quercetin, aucubin, geniposide, geniposidic acid). may regulate key pathways to restore bone balance, offering potential for treating osteoporosis and other aging-related bone diseases

**Objectives:**

This study aims to assess the therapeutic potential of EU in the treatment of aging-related bone diseases.

**Methods:**

A literature search was conducted on the PubMed database up to November 2024 using the search term: “Eucommia AND (bone OR cartilage OR joint).”

**Results:**

The review indicates that EU formulas, extracts, and bioactive components promote osteogenesis, suppress bone resorption, and exert anti-inflammatory and antioxidant properties. These effects contribute positively to the treatment of aging-related bone diseases.

**Conclusion:**

The therapeutic benefits of EU support its development as a promising tool for preventing and treating aging-related bone diseases. These findings provide new research directions to address related health challenges associated with population aging.

## 1 Introduction

Aging-related bone diseases, particularly osteoporosis (OP) and osteoarthritis (OA), are leading causes of disability and reduced quality of life among the elderly. With the global population aging rapidly, these conditions have imposed an enormous socioeconomic burden (Yokota et al., 2024). Although their clinical manifestations differ—OP is characterized by reduced bone mass and increased fracture risk, while OA primarily affects articular cartilage and subchondral bone—they share several underlying age-associated pathophysiological mechanisms (Słupski et al., 2021; Knights et al., 2023). These include impaired bone remodeling, chronic low-grade inflammation, cellular senescence, oxidative stress, and hormonal imbalances. In OP, the imbalance between osteoblast-mediated bone formation and osteoclast-mediated bone resorption leads to progressive bone loss and structural deterioration. In OA, while cartilage degradation is the hallmark feature, the disease also involves subchondral bone sclerosis, osteophyte formation, and aberrant bone remodeling at joint margins. Furthermore, age-related declines in estrogen and other hormones exacerbate skeletal fragility, while the accumulation of senescent cells in bone and joint tissues promotes tissue degeneration through pro-inflammatory and catabolic secretory pathways (Cui et al., 2022; Bi et al., 2024).

Currently, pharmacological treatments for aging-related bone diseases such as OP and OA face major limitations that hinder their long-term efficacy and broad applicability. In OP, antiresorptive agents like bisphosphonates and denosumab effectively reduce bone resorption but fail to fully restore bone quality or stimulate new bone formation. Anabolic therapies, such as parathyroid hormone analogs, promote bone formation but are constrained by high costs, limited treatment duration, and potential safety concerns. Moreover, these drugs primarily target bone remodeling without adequately addressing fundamental contributors such as cellular senescence, chronic inflammation, or oxidative stress (Ramchand and Leder, 2024). In the case of OA, current pharmacological options mainly focus on symptom relief—primarily pain reduction and inflammation control—using nonsteroidal anti-inflammatory drugs (NSAIDs) or corticosteroids, yet they lack disease-modifying effects capable of slowing or reversing joint degeneration (Abramoff and Caldera, 2020). In addition, the long-term use of these medications is associated with adverse effects, which limit their safety in older populations. Taken together, the multifactorial nature and complex pathophysiology of aging-related bone diseases underscore the urgent need for novel therapies that can simultaneously target multiple pathogenic mechanisms while ensuring improved efficacy and safety.

**FIGURE 1:**
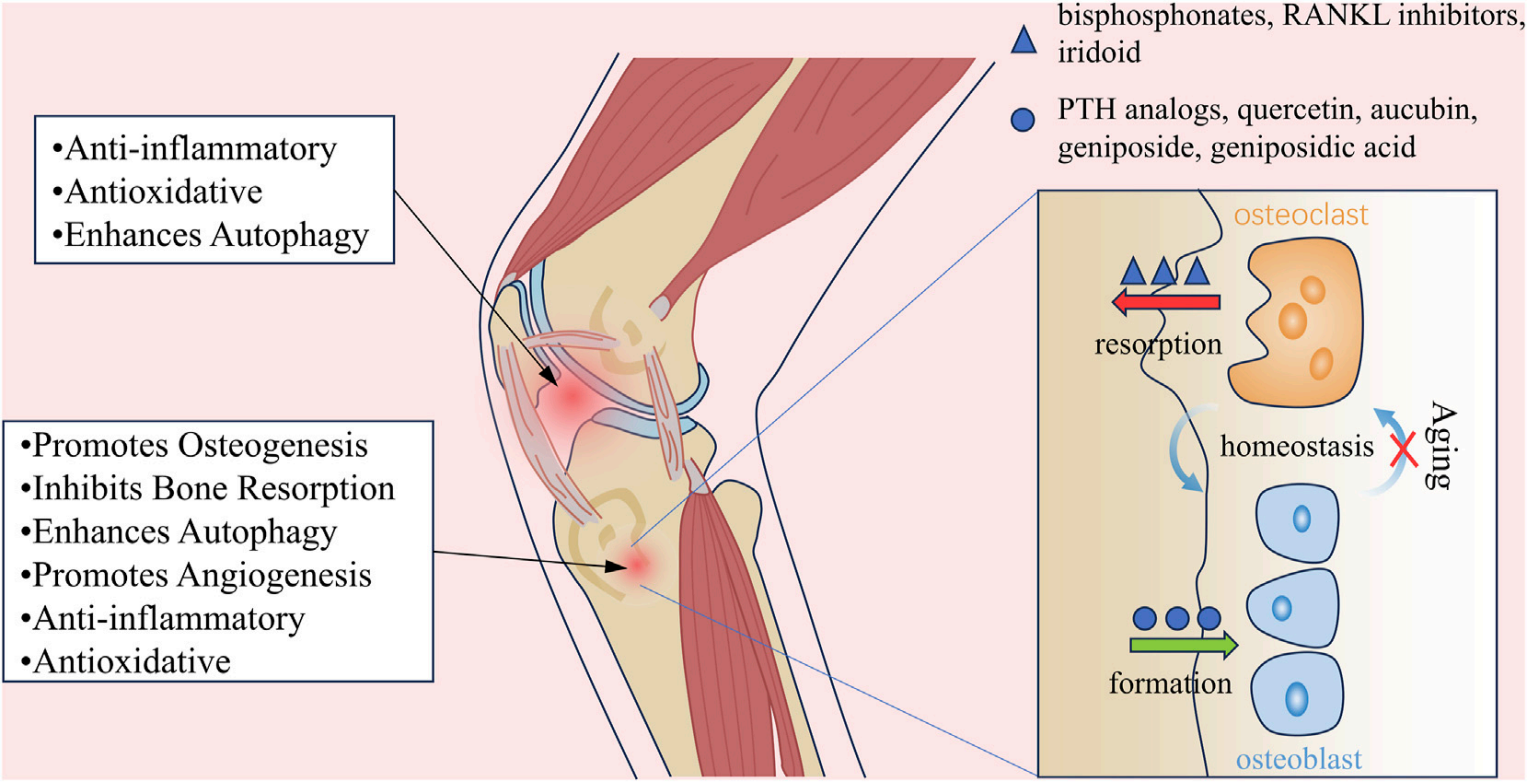
Mechanisms of EU in Treating Aging-related Bone Diseases. (A) EU alleviates aging-related bone diseases through multiple mechanisms. (B) Under physiological conditions, bone homeostasis is maintained by balanced osteoblast-mediated bone formation and osteoclast-driven bone resorption. Aging disrupts this equilibrium, leading to bone loss and structural deterioration. (C) Pharmacological agents restore bone homeostasis through distinct pathways: bisphosphonates and RANKL inhibitors suppress osteoclast-mediated bone resorption, while PTH analogs stimulate osteoblast-driven bone formation. In contrast, bioactive compounds from EU synergistically promote osteogenesis and inhibit resorption, offering a dual-action therapeutic strategy.

*Eucommia ulmoides* Oliv. (EU) is a deciduous tree of the family Eucommiaceae, known for its unique economic and medicinal value, and is widely used in the fields of chemicals, pharmaceuticals, and food industries. EU has a long history of use. Traditionally, its bark was primarily used in traditional Chinese medicine (TCM) and was regarded as both a medicinal and dietary resource (He et al., 2014; Zhu and Sun, 2018). The *Shennong Bencaojing* (*Divine Husbandman’s Classic of Materia Medica*) classifies EU as an “upper herb”, and the *Pharmacopoeia of the People’s Republic of China* states that its effects include “tonifying the liver and kidneys, strengthening the muscles and bones, and preventing miscarriage” (NPC, 2020). For thousands of years, EU has been widely used in TCM to treat symptoms such as lumbar and knee soreness, weakness of the muscles and bones, and fetal restlessness, serving as a core herb in many classic formulas (Wang et al., 2019a; Huang et al., 2021). Furthermore, to fully develop the medicinal value of EU, its bark, flowers, leaves, and seeds are widely used in modern pharmacological research (Figure 1).

Although the overall pharmacological effects of EU have been summarized in reviews, its specific application and mechanism of action in aging-related bone diseases have not been systematically compiled. Therefore, this review will focus on the application and mechanism of EU in aging-related bone diseases in order to analyze its potential medicinal value, provide theoretical support for the modern development of EU, and offer new ideas for exploring the transformation of TCM into modern drugs and addressing the therapeutic challenges of aging-related bone diseases.

## 2 Literature search strategy

**FIGURE 2:**
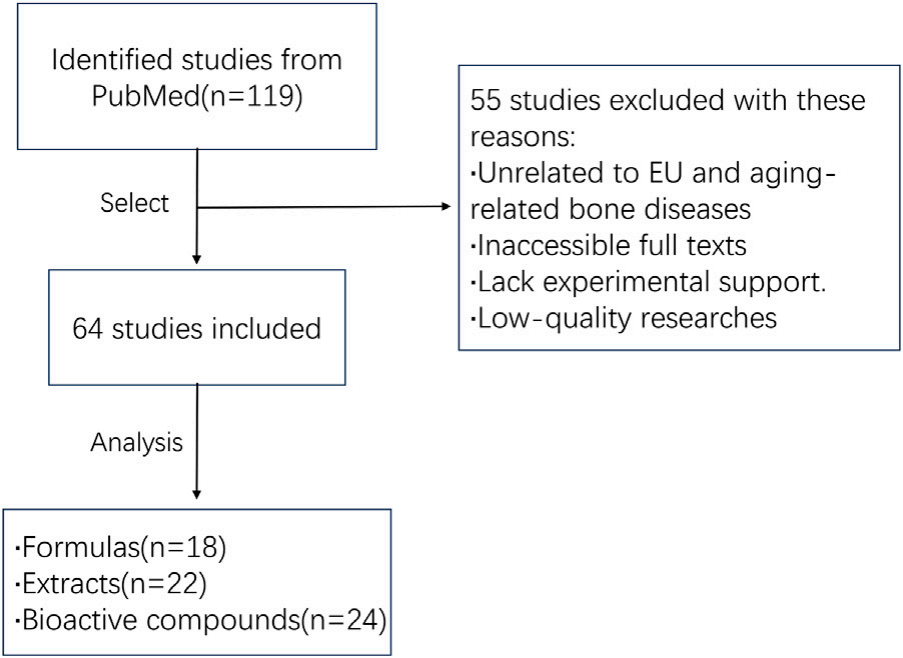
Flow chart of the literature analysis. A total of 119 studies were initially identified from PubMed. After screening, 55 studies were excluded due to irrelevance to EU and aging-related bone diseases, inaccessible full texts, lack of experimental support, or low research quality. Ultimately, 64 eligible studies were included in the review and categorized into three groups: formulas (n = 18), extracts (n = 22), and bioactive compounds (n = 24).

To comprehensively summarize the application and mechanisms of EU in aging-related bone diseases, we performed a systematic literature review with a focus on the effects of EU on the musculoskeletal system. The literature search was based on the PubMed database (www.pubmed.com) and covered all relevant literature from the time of database creation to November 2024. The search terms used were: Eucommia AND (bone OR cartilage OR joint). The initial search yielded 119 publications. After screening and excluding those unrelated to EU and aging-related bone diseases, had inaccessible full texts, had low research quality, or lacked experimental support, 64 studies that met the requirements were finally included (Figure 2).

## 3 Research on EU formulas

An analysis of the retrieved studies revealed that there were more studies on formulas that combined EU with other herbs. These studies have shown that EU formulas fulfill multiple functions, such as promoting osteogenesis, inhibiting bone resorption, anti-inflammation, and regulating muscle metabolism. Table 1 summarizes the results of *in vitro*, *in vivo*, and clinical studies related to the use of EU formulas in the treatment of aging-related bone diseases.

**TABLE 1:** Studies on the treatment of aging-related bone diseases with EU formulas.

Formula composition	Research model	Main mechanisms	Main effects	Reference
Du-Zhong-Wan: Eucommiae Cortex and Radix Dipsaci	OVX rats	Activates estrogen signaling through an estrogen receptor-dependent pathway	Improves BMD, trabecular microarchitecture and biomechanical properties	Li et al. (20216)
	OVX mice with open femoral fracture	Increased expression of the pro-angiogenic factor SLIT3 and promotes H-type vessel angiogenesis at fractured end	Increases bone volume, trabecular number, and bone formation rate, reduces bone erosion area, and promotes healing of osteoporotic fractures	Tian et al. (2022)
Osteo-F: EU, Lycium chinense and Schizandra chinensis	OVX rats	Increases BMP-2 and OPN	Increases BMD	Lee et al. (2021)
Yishen Gushu Formula: EU and other herbs	Ovary-ligated rats	Modulates the TNF-α and IL-17 signaling pathways and reduces TNF-α and IL-1β levels	Increases trabecular thickness and number, and decreases trabecular separation	Liu et al. (2024)
EU, Cuscuta, and Drynaria	Glucocorticoid-induced OP rats	Inhibits the PI3K/Akt signaling pathway	Improves BMD and bone histomorphology	Han et al. (2021)
Eucommiae Cortex and Radix Achyranthis Bidenta	Glucocorticoid-induced OP zebrafish	Upregulates *Runx2*, OP-1, OCN and *β-catenin* levels	Treats glucocorticoid-induced OP	Lee et al. (2022)
Zhuang-Gu-Fang: EU and other herbs	OVX rats	Higher bone formation/resorption ratio, and increases in leptin, ghrelin and PYY levels	Increases BMD, and improves bone structure and osteoblast ultrastructure	Chen et al. (2020)
Eucommiae Cortex, Dipsaci Radix, Achyranthis Bidentatae Radix and Psoraleae Fructus	OA rats developed by anterior cruciate ligament transection followed by treadmill running	Inhibits the expression of p-IKKαβ and COX-2, inhibits NF-κB pathway	Improves OA symptoms and slows down OA progression	Siu et al. (2019)
Ryupunghwan: EU, Astragalus membranaceus, Turnera diffusa, Achyranthes bidentata, Angelica gigas, Eclipta prostrata and Ilex paraguariensis	Human chondrosarcoma cells (SW1353 cells)	Decreased expression of MMP13, collagen II, COX-2, TNF-α, IL-1β and p65	Improves OA symptoms and slows down OA progression	Hong et al. (2018)

### 3.1 Application of EU formulas in OP

EU formulas can be used in the treatment of OP by promoting osteogenesis and inhibiting bone resorption through multiple pathways, which in turn can improve bone mineral density (BMD). For example, *Du-Zhong-Wan* (DZW), a TCM formula made from a 1:1 weight ratio of *Eucommiae Cortex* and *Radix Dipsaci* has shown promising results. Animal studies in ovariectomized (OVX) rats have shown that DZW prevents estrogen deficiency-induced BMD decline by activating estrogen signaling through an estrogen receptor-dependent pathway. Through this pathway, DZW can increase the levels of osteocalcin (OCN) and estradiol (E2), as well as protect trabecular microarchitecture and biomechanical properties (Li et al., 2016). H-type vessels, a newly discovered subtype of skeletal blood vessels, provide essential nutrients to bone tissue and also effectively promote bone formation and bone repair. Research targeting H-vessels offers new directions in the treatment of skeletal diseases (Peng et al., 2020). DZW has been shown to promote H-type vessel angiogenesis at the fractured end by increasing the expression of the pro-angiogenic factor, SLIT3, thereby enhancing osteogenesis and supporting the repair of osteoporotic fractures (Tian et al., 2022). Inflammation plays a key role in many diseases, and OP is no exception (Iantomasi et al., 2023). Broadly targeted plant metabolomics technology, combined with animal experiments, revealed that the *Yishen Gushu Formula* can reduce the expression of pro-inflammatory factors by regulating the TNF-α and IL-17 signaling pathways in postmenopausal osteoporotic rats, thereby increasing trabecular thickness and number, decreasing trabecular separation, and exhibiting excellent osteoprotective effects (Liu et al., 2024). In addition, another formula composed of EU, *Cuscuta*, and *Drynaria* was found to inhibit osteoclast differentiation by suppressing the PI3K/Akt signaling pathway, resulting in improved BMD and bone histomorphology (Han et al., 2021). Interestingly, the mechanism underlying OP treatment by *Zhuang-Gu-Fang*, with EU as the chief herb, appears to be related to the regulation of gut hormones. Administration of *Zhuang-Gu-Fang* to OVX rats led to elevated levels of leptin, ghrelin, and PYY, as well as improved bone microarchitecture (Chen et al., 2020). Although Remmel et al. have also revealed an association between gut hormones and bone mineralization, the exact mechanisms require further investigation (Remmel et al., 2015).

### 3.2 Application of EU formulas in OA

Studies on the treatment of OA with EU formulas have mainly focused on their role in reducing inflammation and protecting cartilage tissue. A retrospective study has demonstrated that the combination of a compound EU bone tonic granules with meloxicam is more effective than meloxicam alone in treating the condition. This combination reduced serum concentrations of IL-17 and S100A12, suppressed inflammation, and significantly alleviated OA symptoms in patients (Hu et al., 2020). In a surgically-induced OA rabbit model, a formula composed of *Eucommiae Cortex*, *Pomegranate*, and *Achyranthis Radix* mixed in a 4:5:1 ratio also exhibited significant anti-inflammatory effects, effectively protecting cartilage tissue (Choi et al., 2020). COX-2, a target of NSAIDs is also a target of EU formulas. In a rat model of OA induced by anterior cruciate ligament transection followed by treadmill running, a combination of *Eucommiae Cortex*, *Dipsaci Radix*, *Achyranthis Bidentatae Radix*, and *Psoraleae Fructus* produced anti-inflammatory and symptom-relieving effects by inhibiting the expression of p-IKKαβ and COX-2 expression, and regulating the NF-κB pathway (Siu et al., 2019). In addition, the *Ryupunghwan* formula, with EU as the chief herb, was also able to decrease COX-2 expression. Surprisingly, the study showed that this herbal formula had no significant effect on COX-1, suggesting fewer gastrointestinal side effects compared to traditional NSAIDs (Hong et al., 2018). These studies indicate that EU formulas have great potential in the treatment of OA, with the advantage of causing fewer side effects than existing drugs. The results provide a strong scientific foundation for the further development of these herbal treatments.

### 3.3 Compatibility and synergistic effects of EU formulas

Studies on the compatibility and synergistic effects of EU remain relatively limited. The *Qing'E Formula*, a TCM formula with EU and *Psoraleae Fructus* as core ingredients, complemented by *Garlic Rhizoma* and *Juglandis Semen*, is commonly used to treat lumbar and knee pain. The estrogen-like effects of *Qing'E Formula* have been confirmed in cellular and animal experiments. Individual studies on EU and *Psoraleae Fructus* have shown their estrogen-like effects, while *Garlic Rhizoma* and *Juglandis Semen* do not produce these effects independently but enhance the estrogen-like effects of EU and *Psoraleae Fructus* when included in the formulae (Xiong et al., 2022). The combination of EU and *Achyranthis Radix* also showed synergistic effects in a zebrafish model of glucocorticoid-induced OP. This formula protected osteoblast function by enhancing the expression of osteogenic genes, such as *Runx2* and *β-catenin*, and exerted the greatest effect at a mass ratio of 1:1 between the two herbs (Lee et al., 2022). These findings further confirm the scientific basis for combining EU with other herbs into a formula for the treatment of aging-related bone diseases while also emphasizing the importance of the synergistic effects among the ingredients in the formula to enhance therapeutic efficacy. By increasing or decreasing the number of herbs or adjusting their proportions, TCM practitioners can change the therapeutic effect of formulas and optimize therapeutic efficacy by flexibly combining herbs for different diseases, thus demonstrating their unique treatment concepts. However, the synergistic mechanisms among herbs are highly complex and have not yet been fully elucidated. Therefore, in-depth research on the rationality of herbal compatibility is of great significance in promoting the modernization of TCM.

## 4 Research on EU extracts

Studies on EU extracts have mainly focused on ethanol and aqueous extracts of *Eucommiae Cortex*, but a few have also examined extracts of EU flowers and leaves. Table 2 summarizes the results of *in vitro* and *in vivo* studies on EU extracts in the treatment of aging-related bone diseases, which have explored their mechanisms of action from various perspectives.

**TABLE 2:** Studies on the treatment of aging-related bone diseases with EU extracts.

EU extracts	Research model	Main mechanisms	Main effects	Reference
EU cortex ethanol extract	Chronic kidney disease mineral bone disorder (CKD-MBD) mice induced by 5/6 nephrectomy combined with low calcium and high phosphorus diet	Activates the PPARG/AMPK signaling pathway	Attenuates renal and bone injuries in CKD-MBD mice	Shen et al. (2023)
	Hind limb suspension-induced disuse OP rats	Upregulates ALP and OCN levels and downregulates TRAP, DPD, CTX and NTX levels	Improves bone microarchitecture and prevents disuse OP	Pan et al. (2014)
	Four-week-old female Sprague-Dawley rats	Promotes cartilage formation and upregulates BMP-2 and IGF-1 levels	Increases longitudinal bone growth rate and growth plate height	Kim et al. (2015)
EU cortex aqueous extract	Diabetic OP mice	Activates the Nrf2/HO-1 signaling pathway, upregulates the expression of TRPV5, PMCA-1b, and CaBP-9k in the intestine and kidney, and upregulates the expression of *Runx*2 and BMP-2 in bone tissue	Lowers blood glucose, reduces oxidative stress, increases calcium absorption, and improves bone microarchitecture and BMD	Shen et al. (2024)
	Rat pituitary cells, osteoblasts and osteoclasts	Induces growth hormone release	Promotes osteoblast proliferation and inhibits osteoclast proliferation	Ha et al. (2003)
	Lipopolysaccharide-stimulated RAW 264.7 macrophages	Reduces NO production, inhibits the PI3K/Akt/mTOR, IFN-β/STAT, NF-κB, MAPK pathways	Reduces inflammation	Koh et al. (2017)
EU flower ethanol extract	Collagen-induced OA rat model	Inhibits the NF-κB pathway, and suppresses the expression of inflammatory factors and pro-angiogenic factors	Inhibits synoviocyte proliferation, suppresses osteoclast differentiation, increases bone mass, and alleviates joint damage	Zhang et al. (2021)
EU leaf ethanol extract	MC3T3-E1 cells	Downregulates the expressions of caspase-3, caspase-6, caspase-7 and caspase-9	Promotes proliferation of MC3T3-E1 cells	Lin et al. (2011)
EU leaf aqueous extract	OVX rats	Increases serum OCN concentrations and decreases DPD and NTX concentrations	Increases BMD, decreases BMI	Zhang et al. (2012)
	Senescence-accelerated mice P6	Increases gut bacterial diversity and increases fecal and serum concentrations of SCFA	Inhibits osteoclast formation	Zhao et al. (2020)
Total glycosides from EU seed	OVX rats	Inhibits the Notch signaling pathway, enhances ALP activity and calcium deposition, increases Osterix, OCN and *Runx*2 levels	Increases trabecular number	Zhou and Xie (2021)
Total lignans	OVX rats	Upregulates OPG levels	Improves BMD, bone microarchitecture and bone biomechanical properties	Zhang et al. (2014)
	OA rabbit induced by anterior cruciate ligament transection	Downregulates IL-6, IL-18 and IL-1β levels, and upregulates BMP-6, arginase-1 and TGF-β levels. Inhibits M1-like macrophage expression and increases M2-like macrophage expression	Reduces cartilage damage and promotes early bone reconstruction	Sun et al. (2021)

### 4.1 Application of EU extracts in OP

Studies have shown that the ethanol extract of EU cortex can treat chronic kidney disease mineral bone disorder (CKD-MBD) by activating the PPARG/AMPK signaling pathway. This activation not only attenuates secondary bone damage but also exerts therapeutic effects on the primary disease (Shen et al., 2023). Similarly, in diabetic OP mice, the aqueous extract of EU cortex reduced oxidative stress by activating the Nrf2/HO-1 signaling pathway, while also increasing renal and intestinal calcium uptake. This resulted in improved BMD, better bone microarchitecture, and alleviation of OP caused by metabolic disorders. Furthermore, the aqueous extract of EU cortex reduced the blood glucose level in the model mice *in vivo* (Shen et al., 2024). EU has also been shown to regulate blood glucose and protect renal function (Park et al., 2006; Li et al., 2021). However, it is unclear whether there is an intrinsic correlation between the efficacy of EU in different diseases. It is commonly accepted that the human body is an organic whole with mutual interactions among different physiological pathways. EU may indirectly affect bone metabolism by modulating kidney function and blood glucose levels. Interestingly, administration of the aqueous extract of EU leaves increased gut microbiota diversity and elevated fecal and serum short-chain fatty acids, which improved OP. This phenomenon indirectly demonstrates the speculation that EU cortex may have systemic modulatory effects (Zhao et al., 2020).

Another study on the aqueous extracts of EU cortex examined the effects of EU on growth hormone. The results showed that the aqueous extract of EU cortex induced the release of growth hormone, which in turn promoted the proliferation of osteoblasts and inhibited the proliferation of osteoclasts. This suggests that EU may play a crucial role in promoting bone metabolism by regulating growth hormone levels (Ha et al., 2003). A Korean study further confirms these findings: administering the ethanol extract of EU cortex to four-week-old rats led to an increase in BMP-2 and IGF-1 levels, as well as an increase in longitudinal bone growth rate and growth plate height (Kim et al., 2015). As IGF-1 mediates growth hormone action, it plays a critical role not only in growth and development but also in bone metabolism (Dixit et al., 2021).

### 4.2 Effects of EU extracts in OA

In rats injected with collagen to induce OA, pannus formation and synovial hyperplasia were observed in the joints, which was reduced by ethanol extract of EU cortex. Serum assays showed a reduction in the expression of inflammatory factors after administration, suggesting that the ethanol extract can attenuate joint inflammation by decreasing the levels of inflammatory factors (Xing et al., 2020). Further studies suggest that this effect may be related to the PI3K/Akt signaling pathway. By inhibiting this pathway, the aqueous extract of EU cortex reduces the expression of inflammatory factors, while also decreasing the secretion of matrix metalloproteinases such as MMP-3, thereby protecting cartilage tissue (Xie et al., 2015). In addition, the aqueous extract of EU cortex can exert anti-inflammatory effects by inhibiting the IFN-β/STAT, NF-κB, and MAPK pathways. This not only delays OA progression but also offers the possibility of articular cartilage repair (Koh et al., 2017).

### 4.3 Effects of EU extracts in RA

Limited evidence has elucidated the multi-target therapeutic mechanisms of EU in the treatment of rheumatoid arthritis (RA). Based on network pharmacology, the pharmacological mechanisms of EU in treating RA have been predicted, revealing that EU may exert its effects through pathways such as the TNF pathway and the IL-17 pathway (Ying et al., 2022). The findings from the cell and animal studies by Wang et al. provide further validation for the therapeutic potential of EU in treating RA. Specifically, the 70% ethanol extract of EU demonstrates a multifaceted therapeutic effect on RA through a series of interrelated mechanisms. It effectively inhibits synovial hyperplasia, thereby reducing the proliferation of inflamed synovial cells. This action is complemented by its ability to lower the population of Th17 cells and the corresponding levels of serum IL-17, while simultaneously enhancing the IL-10-mediated anti-inflammatory response. Additionally, the extract suppresses the production of TNF-α and IL-1β in both serum and tissues, ultimately mitigating cartilage and bone degradation. These mechanisms work in concert to alleviate RA symptoms comprehensively (Wang et al., 2016). Notably, the iridoid components of EU modulate and attenuate the invasion/migration of HFLS-RA cells through the JAK2/STAT3 pathway, which is evidenced by the decreased phosphorylation of p-JAK2/p-STAT3 and the downregulation of inflammatory genes (Tang et al., 2023). Furthermore, the ethanol extract of EU male flowers exhibits dose-dependent inhibitory effects on synovial proliferation by suppressing the NF-κB pathway, accompanied by pro-apoptotic effects. In collagen-induced arthritis (CIA) models, this intervention reduces osteoclast differentiation, joint inflammation, and the expression of angiogenic factors, while also delaying structural joint damage (Zhang et al., 2021). Collectively, these findings position EU as a pleiotropic therapeutic agent with the potential to address the inflammatory cascades and tissue remodeling issues in the pathogenesis of RA.

## 5 Research on the bioactive components of EU

**FIGURE 3:**
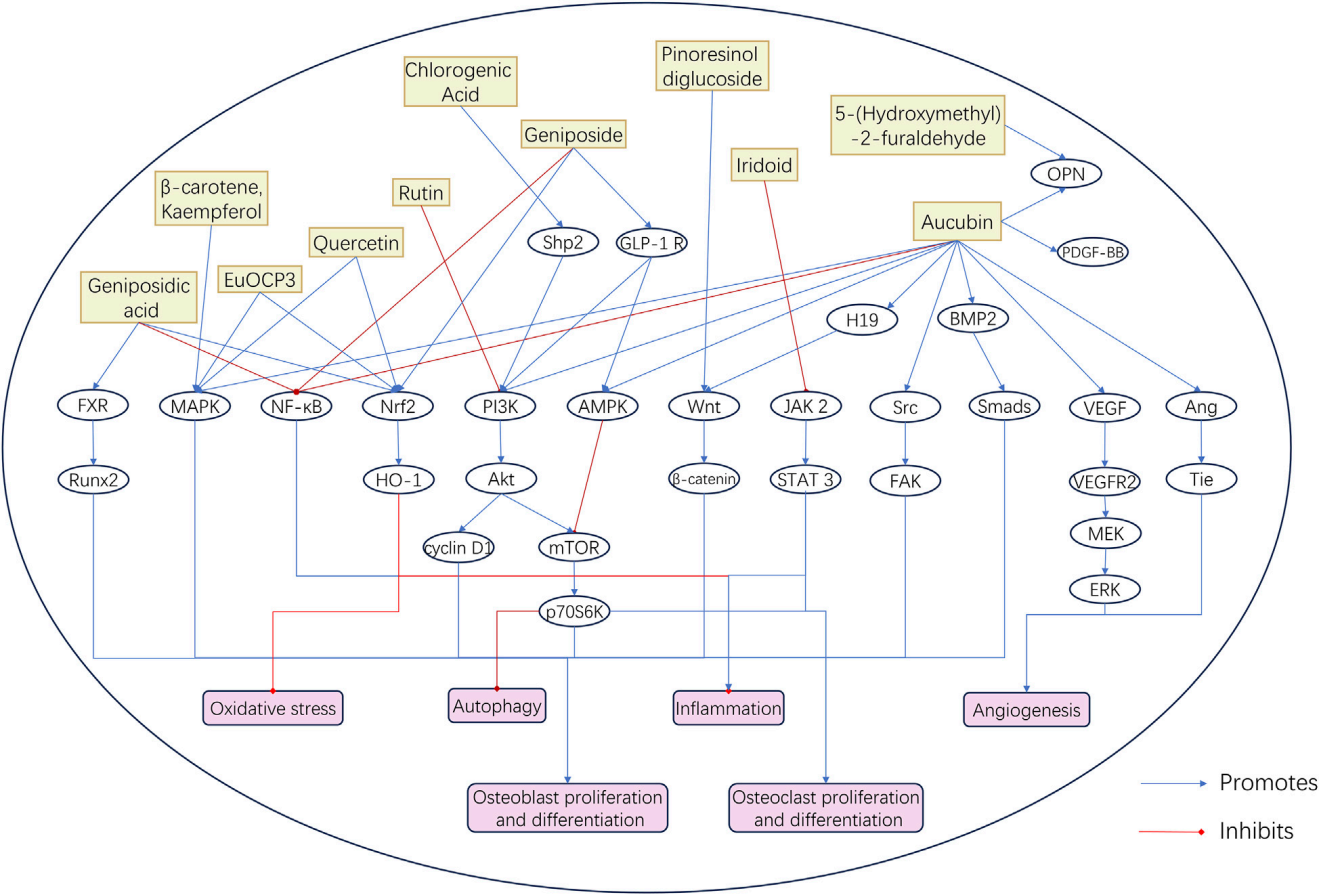
Molecular Mechanism Diagram of Bioactive Components in EU. This schematic diagram illustrates the regulatory effects of representative bioactive compounds in EU—including aucubin, geniposidic acid, chlorogenic acid, rutin, quercetin, geniposide, and iridoids—on key signaling pathways related to bone and joint health. These components modulate pathways such as BMP2/Smads, Wnt/β-catenin, PI3K/Akt/mTOR, MAPK, and NF-κB, thereby promoting osteoblast proliferation and differentiation, enhancing angiogenesis, and inhibiting oxidative stress, inflammation, and autophagy. Blue arrows indicate promotion, and red lines indicate inhibition.

Studies on the bioactive components of EU have mainly focused on their mechanisms of action in treating OP. Among these components, quercetin, aucubin, geniposide, and geniposidic acid are the most promising active ingredients. These components have shown significant biological activity in the regulation of bone metabolism and anti-inflammatory and antioxidant effects. Table 3 summarizes the results of *in vitro* and *in vivo* studies on EU bioactive components in the treatment of aging-related bone diseases. These studies have examined the molecular mechanisms of EU bioactive components in the treatment of aging-related bone diseases from different perspectives (Figure 3).

**TABLE 3:** Studies on the bioactive components of EU in the treatment of aging-related bone diseases.

Bioactive components of EU	Research model	Main mechanisms	Main effects	Reference
Quercetin	Iron overload mouse model induced by injecting iron dextrose intraperitoneally	Activates the Nrf2/HO-1 signaling pathway, downregulates caspase-3 and BAX expression, and upregulates BCL-2 expression	Reduces iron deposition and attenuates bone loss	Xiao et al. (2023a)
	Human nucleus pulposus cells	Reduces PPARA levels	Delays intervertebral disc degeneration	Xu et al. (2023)
Aucubin	Double transgenic medaka with OP induced by overexpressing RANKL after heat-shock treatment, and VEGF tyrosine kinase inhibitor II-induced vascular insufficient transgenic zebrafish model	Upregulates the VEGF/VEGFR2/MEK/ERK, Akt/mTOR, Src/FAK, and Ang/Tie signaling pathways	Suppresses bone resorption and promotes angiogenesis	He et al. (2023)
	OVX mice	Inhibits the MAPK and NF-κB signaling pathways, and increases PDGF-BB production	Promotes H-type vessel angiogenesis, inhibits osteoclast maturation, and attenuates bone loss	Li et al. (2022)
	Dexamethasone-induced OP mice	Promotes synthesis of arachidonic acid into prostaglandin A2 (PGA2)	Increases BMD and improves bone microarchitecture	Wang et al. (2024a)
	MG63 cells	Activates the BMP2-mediated Smads, MAPK and Akt/mTOR/p70S6K signaling pathways	Promotes osteogenic differentiation	Li et al. (2018)
	Dexamethasone-induced MC3T3-E1 cells	Activates the AMPK signaling pathway and enhances autophagy	Inhibits osteoblast apoptosis	Yue et al. (2021)
	H19 knockdown bone marrow mesenchymal stem cells (BMSCs), femur fracture mice	Activates the Wnt/*β-catenin* signaling by promoting H19 expression	Promotes osteogenic differentiation and fracture healing	Mai et al. (2024)
	Surgically-induced OA mice	Inhibits BAX, caspase-3 and caspase-9 expression, promotes BCL-2 expression, and inhibits ROS production	Protects articular cartilage and delays OA progression	Wang et al. (2019b)
Geniposide	High-fat diet-induced OP rats	Activates the Nrf2 pathway and inhibits the NF-κB pathway	Reduces osteoblast apoptosis, improves BMD and bone microarchitecture	Xiao et al. (2023b)
	Dexamethasone-induced OP rats	Activates the GLP-1R/PI3K/Akt/mTOR signaling pathway	Decreases osteoblast apoptosis and increases BMD and trabecular number	Huang et al. (2022)
	OA rats induced by monosodium iodoacetate	Activates the GLP-1R/AMPK/mTOR signaling pathway	Promotes autophagy and protects chondrocytes	Huang et al. (2023)
Geniposidic acid	OVX rats	Activates the FXR/*Runx*2 signaling pathway	Enhances osteoblast activity and increases bone mass	Liu et al. (2023)
	OVX mice, Fxr knockout (^Fxr−/−^) mice and cell models	Activates the FXR/*Runx*2 signaling pathway	Promotes osteogenesis	Liu et al. (2022)
	Nrf2 knockdown chondrocytes, surgically-induced OA rats	Activates the Nrf2 signaling pathway and inhibits the NF-κB signaling pathway	Inhibits inflammation and chondrocyte ferroptosis to protect articular cartilage	Sun et al. (2024)
EuOCP3	Dexamethasone-induced OP mice	Regulates the abundance of specific species in gut microbiota, and activates the Nrf2 signaling pathway	Increases osteoblasts, decreases osteoclasts, and increases cortical bone thickness and mineralized bone area	Song et al. (2023)
Pinoresinol diglucoside	Dexamethasone-induced OP zebrafish	Activates the Wnt/*β-catenin* signaling pathway	Improves OP symptoms and chondrodysplasia	Zuo et al. (2024)
Chlorogenic Acid	OVX rats	Activates the Shp2/PI3K/Akt/cyclin D1 signaling pathway	Promotes osteogenic differentiation and improves BMD and bone microarchitecture	Zhou et al. (2016)
Rutin	OVX rats	Inhibits the Akt/mTOR signaling pathway and downregulates FNDC1 levels	Improves BMD and bone microarchitecture	Xiao et al. (2019)
β-carotene	MC3T3-E1 cells	Activates the MAPK signaling pathway	Promotes osteoblast proliferation and differentiation	Zhou and Wu (2022)
Kaempferol	BMSCs	Increases ALP activity and calcium deposition, upregulates osteogenic marker levels, and decreases caveolin-1 levels	Promotes osteogenic differentiation	Li et al. (2024)
	MC3T3-E1 cells	Activates the JNK signaling pathway	Promotes osteoblast proliferation and differentiation	Zhou and Wu (2022)
5-(Hydroxymethyl)-2-furaldehyde	BMSCs	Promotes Col1-α1, OCN and OPN expression	Promotes osteogenic differentiation and bone mineralization	Tan et al. (2014)
Iridoid	Collagen-induced OA rats, TNF-α-induced HFLS-RA cells	Inhibits HFLS-RA cell invasion and migration, inhibits the JAK2/STAT3 pathway, and prevents CD4^+^ T cell differentiation into Th17 cells	Reduces osteoclasts, reduces joint inflammation and protects joints	Tang et al. (2023)

### 5.1 Quercetin

Quercetin is a flavonoid widely found in fruits, vegetables, and a variety of herbs. It is well known for its excellent antioxidant, antiviral, antibacterial, and anti-inflammatory properties, as well as its good regulatory effects on blood glucose, blood pressure, and lipids. These effects have been extensively reviewed from different perspectives (Di Petrillo et al., 2022; Qi et al., 2022; Hosseini et al., 2021). As one of the main active ingredients in EU, quercetin not only possesses these properties but has also shown to be effective in the treatment of OP. In an iron overload-induced OP mouse model, quercetin inhibited reactive oxygen species (ROS) production by activating the Nrf2/HO-1 signaling pathway, thereby significantly attenuating oxidative stress and reducing osteoblast apoptosis. Additionally, quercetin further protects bone tissue from oxidative damage by regulating the expression of anti-apoptotic (e.g., BCL-2) and pro-apoptotic factors (e.g., caspase-3 and BAX) (Xiao et al., 2023). Quercetin also activates the MAPK1/ERK2 signaling pathway, which promotes osteoblast proliferation and differentiation, highlighting its potential as a natural anti-OP agent (Zhou and Wu, 2022). Furthermore, by reducing PPARA levels, quercetin can delay intervertebral disc degeneration (Xu et al., 2023). However, the clinical application of quercetin is limited by poor bioavailability. Hence, further improvements are needed to increase its practical efficacy, and some progress has been achieved in the research on quercetin derivatives (Alizadeh and Ebrahimzadeh, 2022).

### 5.2 Aucubin

Aucubin is an iridoid glycoside that has garnered significant interest due to its wide range of pharmacological effects. Modern pharmacological studies have found that aucubin has antioxidant, anti-inflammatory, anti-tumor, neuroprotective, and osteoprotective properties (Zeng et al., 2020). In the treatment of OP, aucubin promotes angiogenesis and improves bone metabolism by modulating the VEGF/VEGFR and Ang/Tie signaling pathways (He et al., 2023). The AMPK pathway is another pathway of action for aucubin. By activating the AMPK pathway, aucubin enhances cellular autophagy, thereby inhibiting dexamethasone-induced apoptosis in osteoblasts (Yue et al., 2021). Using untargeted metabolomics techniques, Wang et al. found that arachidonic acid may play a key role in aucubin treatment of Glucocorticoid-induced OP. Further Western blot and RT-qPCR assays showed that aucubin promotes the metabolism of arachidonic acid to produce PGA2, which support bone synthesis (Wang et al., 2024). Aucubin also exerts a protective effect on articular cartilage. In mice with OA induced by meniscal ligament transection, aucubicin inhibited the expression of pro-apoptotic factors (e.g., BAX, caspase-9, and caspase-3), increased BCL-2 expression, and reduced ROS production (Wang et al., 2019b).

### 5.3 Geniposide

Geniposide, another iridoid glycoside, exhibits a range of pharmacological effects, including anti-inflammatory, antidiabetic, antioxidant, neuroprotective, hepatoprotective, and choleretic activities (Shan et al., 2017). In OP, geniposide acts via multiple pathways. It can activate the Nrf2 pathway and reduce oxidized low-density lipoprotein-induced osteoblast apoptosis by downregulating the NF-κB pathway (Xiao et al., 2023). The study revealed that geniposide exerts dual efficacy in the treatment of OP and OA by promoting autophagy, which not only increases BMD and trabecular number, but also protects chondrocytes (Huang et al., 2023; Huang et al., 2022). Given its ability to modulate multiple pathological processes, geniposide may represent a multifunctional therapeutic agent in managing aging-related bone diseases. The integration of autophagy enhancement and anti-inflammatory activity makes it especially valuable for complex conditions like OP coexisting with OA. Further investigations are needed to clarify its long-term efficacy, potential synergism with current treatments, and applicability in clinical settings.

### 5.4 Geniposidic acid

Geniposidic acid, another iridoid compound, is known for its antioxidant and anti-inflammatory properties, and has been experimentally demonstrated to have an ameliorative effect on diseases such as renal fibrosis, cholestatic hepatitis, and colitis (Wang et al., 2024; Song et al., 2022; Jiang et al., 2023). Sun et al. examined the mechanism of geniposidic acid in the treatment of OA and found that it acts by activating the Nrf2 signaling pathway and inhibiting the activation of NF-κB (Sun et al., 2024). It shares similarities with the mechanism of geniposide in the treatment of OP, but further research is needed to see if both compounds treat OP and OA through the same mechanism. Two other studies demonstrated the ability of geniposidic acid to activate the FXR/*Runx*2 signaling pathway, which promotes osteogenesis. Importantly, in Fxr-knockout rats, geniposidic acid failed to upregulate *Runx*2 or promote osteogenesis, indicating that its osteogenic effects depend on FXR signaling (Liu et al., 2023; Liu et al., 2022). However, the potential link between the three signaling pathways (FXR, Nrf2, and NF-κB), and the specific mechanism of action for geniposidic acid, remain unclear. Notably, geniposidic acid enters Caco-2 cells by passive diffusion, but salt treatments enhance its cellular uptake, suggesting that preparation methods may influence EU efficacy (Lu et al., 2018). This processing-dependent alteration in bioactive compound bioavailability underscores the imperative for mechanistic investigations to optimize the therapeutic outcomes and safety profiles of botanical preparations.

## 6 Discussion

This review synthesizes recent advances in the application of EU formulas, extracts, and bioactive compounds for treating aging-related bone diseases. Accumulated evidence demonstrates that EU exerts multifaceted therapeutic effects including pro-osteogenic, anti-resorptive, anti-inflammatory, and antioxidant actions through modulation of key signaling pathways such as Wnt/β-catenin, BMP/Smad, and JAK/STAT, thereby restoring bone homeostasis. While OVX rat models remain predominant in pharmacological evaluations, innovative approaches like transgenic zebrafish models coupled with real-time imaging have provided new insights into EU’s skeletal and vascular interactions (He et al., 2023). Emerging interdisciplinary strategies further enhance EU’s potential: surface modification of polyetheretherketone implants with EU polysaccharides and strontium synergistically improves osteointegration (Mengdi et al., 2022), and osteoblast-targeting delivery systems enable precise transport of geniposidic acid to bone-forming cells, addressing bioavailability challenges in natural product utilization (Liu et al., 2023).

Toxicological assessments consistently show that EU extracts have an excellent safety profile. Cytotoxicity assays revealed no inhibitory effects on RA-FLS cells even at high concentrations (up to 1,000 μg/mL) for ethanol extracts of EU bark, leaves, and male flowers., confirming negligible cellular toxicity (Xing et al., 2020). Furthermore, in drug intervention models, both low-dose (200 mg/kg) EU and salt-processed EU groups demonstrated significant mitigation of renal pathologies compared to controls, including reduced renal calcification, connective tissue hyperplasia, interstitial fibrosis, tubular ectasia, and lymphocyte infiltration. Additionally, the high-dose (600 mg/kg) EU and salt-processed EU groups exhibited enhanced therapeutic effects (Shen et al., 2023). Similar outcomes were also observed in another study (Wu et al., 2024). Crucially, EU’s phytoestrogenic properties mimic bone-protective estrogenic activity while circumventing endometrial carcinogenesis risks associated with conventional hormone replacement therapies (Zhang et al., 2014). These breakthroughs open up new possibilities for the transformation of EU from a traditional herb to a modern, mechanism-driven therapeutic agent for bone diseases.

Despite notable advances, several limitations persist. The precise mechanisms through which EU exerts its therapeutic effects on aging-related bone diseases remain incompletely understood. Current research has predominantly centered on OP and OA, while investigations into other conditions such as RA are comparatively scarce. Importantly, EU may offer broader systemic benefits beyond skeletal protection, owing to its anti-inflammatory, antioxidant, and anti-apoptotic properties. These actions are not only critical for maintaining bone homeostasis but are also relevant to a spectrum of aging-related comorbidities, including sarcopenia, frailty, cardiovascular diseases, and neurodegenerative disorders. Notably, chronic low-grade inflammation and oxidative stress are recognized as common pathological drivers across these conditions (Zuo et al., 2019). Therefore, the multi-targeted effects of EU—mediated via pathways such as Nrf2, AMPK, NF-κB, and JAK/STAT—may confer synergistic benefits, both in mitigating bone degeneration and addressing systemic aging processes. From a comprehensive and aging-focused perspective, interventions based in the EU could help not only with treating OP and OA but also with slowing down the loss of physical function and improving overall health in older adults. This means that the EU could play a key role in developing holistic approaches that go beyond treating specific organs and instead support the broader goal of healthy aging.

Additionally, EU holds promise in combination therapy. Given its multi-component and multi-targeted pharmacological profile, EU appears particularly suitable as an adjunct to modern pharmacotherapy. Its anti-inflammatory, antioxidant, and osteogenic activities suggest potential synergy with conventional agents. For instance, co-administration with bisphosphonates—the first-line treatment for OP—might enhance therapeutic outcomes while potentially mitigating long-term adverse effects such as atypical fractures and gastrointestinal discomfort. Likewise, in OA management, EU’s anti-inflammatory actions could allow for dose reduction of NSAIDs, thereby lowering the risks of cardiovascular and gastrointestinal complications. Although direct evidence remains limited, these hypothetical benefits warrant further investigation in preclinical and clinical studies.

In addition, current studies on EU’s bioactive compounds are limited in number and scope. Most findings are derived from preliminary preclinical models, often focusing on single signaling pathways or disease types. There is a lack of systematic investigation into their bioavailability, pharmacokinetics, long-term efficacy, and synergistic interactions within the bioactive compounds of EU. Moreover, their therapeutic potential in clinical settings has not yet been validated.

Therefore, future studies should place greater emphasis on elucidating the molecular mechanisms of EU and its bioactive constituents, as well as conducting rigorous clinical validation across a broader spectrum of aging-related bone diseases. Moreover, enhancing the bioavailability and physicochemical stability of these active ingredients is essential to fully realize their therapeutic potential. Research should actively explore bioavailability-enhancing strategies, including the use of nanocarriers, liposomal encapsulation, prodrug design, and co-administration with absorption enhancers. These advancements are critical for bridging the gap between promising preclinical findings and effective clinical application.

## 7 Conclusion

In summary, mounting evidence supports EU and its bioactive components as promising candidates for the prevention and treatment of aging-related bone diseases. These therapeutic effects are driven not by single agents but through the synergistic regulation of multiple signaling pathways by a diverse array of phytochemicals.

Based on current knowledge, we hypothesize that the osteoprotective actions of EU result from its ability to modulate multiple interconnected biological networks, contributing not only to bone regeneration but also to the alleviation of aging-associated systemic disorders. Future research should focus on elucidating the integrated mechanisms of EU’s action, validating clinical efficacy, and optimizing bioavailability and formulation stability.

In addition, exploring combination therapies that pair EU with other TCM herbs or modern pharmaceuticals may provide novel, safer, and more effective therapeutic regimens—particularly for older adults with comorbidities. Such efforts are essential for transforming EU from a traditional herbal remedy into a modern, mechanism-driven therapeutic agent capable of addressing the complex challenges of musculoskeletal aging and promoting healthy longevity.

## Glossary

Akt: protein kinase B
ALP: alkaline phosphatase
AMPK: adenosine monophosphate-activated protein kinase
Ang: angiopoietin
BAX: BCL-2-associated X protein
BCL-2: B-cell lymphoma-2
BMD: bone mineral density
BMP: bone morphogenetic protein
BMSCs: bone marrow mesenchymal stem cells
CaBP-9k: calbindin-D9k
CTX: C-terminal crosslinked telopeptides of collagen type I
DPD: urinary deoxypyridinoline
ERK: extracellular signal-regulated kinase
FAK: focal adhesion kinase
FNDC1: fibronectin type iii domain containing 1
FXR: Farnesoid X receptor
GLP-1R: glucagon-like peptide-1 receptor
HFLS-RA: human fibroblast-like synoviocytes from rheumatoid arthritis donors
HO-1: heme oxygenase 1
IFN-β: interferon-β
IGF: insulin-like growth factors
IL: interleukin
JAK2: Janus Kinase 2
JNK: c- Jun N-terminal kinase
MAPK: mitogen-activated protein kinase
**MEK**,: mitogen-activated protein kinase
MMP: matrix metalloproteinases
mTOR: mammalian target of rapamycin
NF-κB: nuclear factor κ-light-chain-enhancer of activated B cells
Nrf2: nuclear factor erythroid 2-related factor 2
NTX: N-terminal crosslinked telopeptides of collagen type I
OCN: osteocalcin
OPG: osteoprotegerin
OPN: osteopontin
OP-1: osteogenic protein-1
OVX: ovariectomized
p-IKKαβ: phosphorylated IκB kinase α and β
PDGF-BB: platelet-derived growth factor-BB
PGA2: prostaglandin A2
PI3K: phosphoinositide 3-kinase
PMCA-1b: plasma membrane calcium-ATPase 1b
PPARA: peroxisome proliferator-activated receptor alpha
PPARG: Peroxisome Proliferator-activated Receptor Gamma
PYY: peptide YY
ROS: reactive oxygen species
Runx2: runt-related transcription factor 2
SCFA: short-chain fatty acids
Shp2: Src homology 2-containing phosphatase 2
SLIT3: slit guidance ligand 3
Src: Src family of kinases
STAT: signal transducer and activator of transcription
S100A12: S100 calcium binding protein A12
TGF-β: transforming growth factor-β
Tie: tyrosine kinase with immunoglobulin and EGF homology domains
TNF-α: tumor necrosis factor-α
TRAP: tartrate resistant acid phosphatase
TRPV5: transient receptor potential vanilloid 5
VEGF: vascular endothelial growth factor
VEGFR2: vascular endothelial growth factor receptor 2
